# Assessing the Partial Hessian Approximation in QM/MM-Based Vibrational Analysis

**DOI:** 10.1021/acs.jctc.4c00882

**Published:** 2024-10-18

**Authors:** Jonas Vester, Jógvan Magnus Haugaard Olsen

**Affiliations:** † DTU Chemistry, Technical University of Denmark, DK-2800 Kgs. Lyngby, Denmark; ‡ Hylleraas Centre for Quantum Molecular Sciences, Department of Chemistry, UiT The Arctic University of Norway, N-9037 Tromsø, Norway

## Abstract

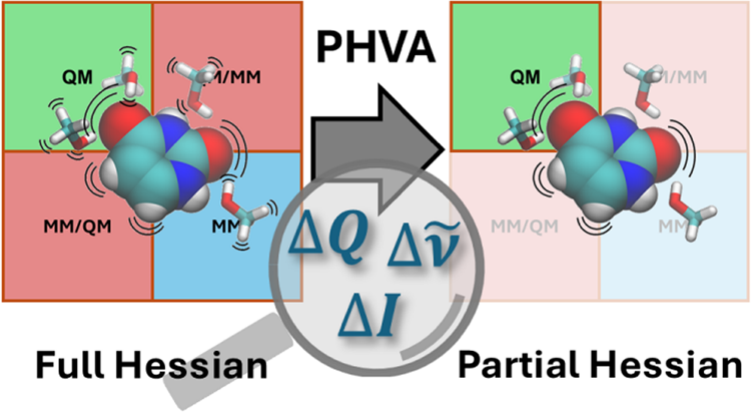

The partial Hessian approximation is often used in vibrational analysis of quantum mechanics/molecular mechanics (QM/MM) systems because calculating the full Hessian matrix is computationally impractical. This approach aligns with the core concept of QM/MM, which focuses on the QM subsystem. Thus, using the partial Hessian approximation implies that the main interest is in the local vibrational modes of the QM subsystem. Here, we investigate the accuracy and applicability of the partial Hessian vibrational analysis (PHVA) approach as it is typically used within QM/MM, i.e., only the Hessian belonging to the QM subsystem is computed. We focus on solute–solvent systems with small, rigid solutes. To separate two of the major sources of errors, we perform two separate analyses. First, we study the effects of the partial Hessian approximation on local normal modes, harmonic frequencies, and harmonic IR and Raman intensities by comparing them to those obtained using full Hessians, where both partial and full Hessians are calculated at the QM level. Then, we quantify the errors introduced by QM/MM used with the PHVA by comparing normal modes, frequencies, and intensities obtained using partial Hessians calculated using a QM/MM-type embedding approach to those obtained using partial Hessians calculated at the QM level. Another aspect of the PHVA is the appearance of normal modes resembling the translation and rotation of the QM subsystem. These pseudotranslational and pseudorotational modes should be removed as they are collective vibrations of the atoms in the QM subsystem relative to a frozen MM subsystem and, thus, not well-described. We show that projecting out translation and rotation, usually done for systems in isolation, can adversely affect other normal modes. Instead, the pseudotranslational and pseudorotational modes can be identified and removed.

## 1 Introduction

The simulation of large molecular systems poses substantial challenges that are particularly significant when quantum mechanical modeling is required, such as in calculations or simulations involving chemical reactions or light–matter interactions. Computational methods rooted in molecular quantum mechanics are limited to relatively small systems due to the high computational cost. To tackle this issue, the common strategy is to employ a multiscale quantum mechanics/molecular mechanics (QM/MM) approach.^1−5^ Here, the system is partitioned into two subsystems: the region of interest (the QM subsystem), treated quantum mechanically, and the surrounding environment (the MM subsystem), described using classical molecular mechanics. This approach has the benefit of preserving the atomistic structure of the environment, which is crucial for accurately representing directional and structural influences on, e.g., molecular properties. The QM/MM approach enables the calculation of local molecular properties, including vibrational properties that provide valuable insights into the chemical structure, bonding characteristics, and intermolecular interactions.^6,7^

There are two main computational approaches for simulating vibrational spectra. In the dynamic approach, spectra are obtained from Fourier-transformed time-correlation functions of relevant properties, e.g., dipoles for infrared (IR) and polarizabilities for Raman spectra, obtained through molecular dynamics (MD) simulations.^8−10^ In the static approach, known as vibrational analysis, vibrational frequencies and normal modes are obtained from an eigenanalysis of the molecular Hessian and the associated intensities are calculated using geometrical derivatives of the relevant properties.^11^ The dynamic approach includes temperature effects and anharmonicity for the QM subsystem but may require a relatively large number of property calculations. In contrast, a vibrational analysis is performed on geometry-optimized structures, thus requiring fewer calculations, i.e., one per conformer. However, it does not include temperature effects and requires more elaborate calculations to include anharmonicity.^12,13^ In this work, we focus on the vibrational analysis approach as part of a larger effort to develop an efficient approach to simulate vibrational spectra of large and complex molecular systems.

Performing vibrational analysis of large QM/MM systems is impractical due to the computational cost of calculating the full Hessian, mainly the computationally expensive derivatives with respect to the positions of the nuclei in the QM subsystem. One way to reduce the computational cost is the partial Hessian vibrational analysis (PHVA) approximation.^14−16^ Using PHVA within QM/MM^17−19^ usually implies that only geometrical derivatives with respect to QM nuclei are included, i.e., only the QM–QM block of the Hessian matrix is calculated, disregarding the MM–MM, QM–MM, and MM–QM blocks. The outcome is a system with mobile QM atoms surrounded by immobile MM atoms. Here, we will refer to this approach as QM/MM-PHVA. Other more advanced partial Hessian approaches have been proposed, such as the mobile block Hessian (MBH)^20−22^ and the vibrational subsystem analysis (VSA)^23−25^ approaches. In this work, we only consider the PHVA. Specifically, we will use it with the fragment-based polarizable embedding (PE) model^26,27^ extended recently to enable calculations of analytical Hessians and geometrical property derivatives.^28^ The fragment-based PE approach is one of several PE models that implement analytic Hessians using an atomistic polarizable environment.^28−33^

The eigenanalysis of the partial Hessian in the QM/MM-PHVA approach results in 3*N* normal modes and corresponding frequencies (where *N* is the number of atoms in the QM subsystem). A minimum of six normal modes (assuming a nonlinear molecule) correspond to vibrations that can be characterized as full or partial pseudotranslational and pseudorotational modes because they resemble translations and rotations of the QM subsystem in a vacuum. These modes would involve atoms in both the QM and MM subsystems in the full Hessian vibrational analysis, but because the MM atoms are immobile by design, it is only the QM atoms that are collectively vibrating inside a cage defined by the MM atoms. Pseudotranslational and pseudorotational modes are low-frequency but not necessarily the lowest ones, and moreover, more than six modes may have pseudotranslational and/or pseudorotational character. These normal modes are not well described, and there is thus a need to identify those most affected so they can be filtered out. The practice of projecting out translation and rotation as part of the vibrational analysis for molecules in a vacuum is invalid within the PHVA approximation. Instead, we introduce a simple scheme to identify the problematic modes and quantify the pseudotranslational and/or pseudorotational character.

The PHVA approach, using pure QM and MM methods, has shown promising results for local modes.^34−37^ These studies are encouraging for the focused QM/MM-PHVA approach, which also targets local modes within the QM subsystem. In our study, we assess the QM/MM-PHVA method, applying it to solute–solvent systems with small, rigid solutes. We also investigate the impact of expanding the QM subsystem by including selected solvent molecules. Our goal is to evaluate the errors introduced by the QM/MM-PHVA approximation on harmonic frequencies and the corresponding IR and Raman intensities. To achieve this, we computed full Hessians, dipole gradients, and polarizability gradients based on full QM calculations for several model systems. We can assess the errors from the PHVA by comparing the partial Hessian and property gradients to the full quantities. Subsequently, we will analyze the discrepancies introduced by the QM/MM approach by comparing partial Hessians obtained from full QM calculations to those derived from QM/MM calculations.

## 2 Methods

### 2.1 Computational Details

We use seven solute–solvent systems to investigate the errors introduced by the QM/MM-PHVA approach on normal modes, frequencies, IR intensities, and Raman intensities. The systems consist of single solute molecules surrounded by multiple solvent molecules: furan in benzene, naphthalene in acetonitrile, pyridine in methanol, 1,3-butadiene in water, uracil in methanol, propanamide in water, and formaldehyde in water. The starting structures were obtained from Reinholdt et al.^38^ First, we extracted spheres with an 8 Å radius, where the center of mass of the solutes is at the center of the spheres. Preliminary calculations indicated that using an 8 Å radius yields similar frequencies and intensities compared to larger radii, up to 24 Å. Furthermore, the exact system size is not critically important since these structures are used for internal comparison. We also prepared small model systems of pyridine in methanol, 1,3-butadiene in water, uracil in methanol, propanamide in water, and formaldehyde in water by removing most solvent molecules. Thus, between 4 and 11 solvent molecules surrounding the solute molecules were kept (more details in Table 1).

**Table 1:** List of Investigated Solute–Solvent Systems

	number of solvent molecules	
system	large	small
furan in benzene	15	
naphthalene in acetonitrile	19	
pyridine in methanol	30	6
1,3-butadiene in water	65	7
uracil in methanol	32	4
propanamide in water	71	6
formaldehyde in water	86	11

The HF/pcseg-1^39^ level of theory was used for the full QM calculations on the small model systems. For the QM/MM calculations on the large systems and the small model systems, the solutes were also modeled at the HF/pcseg-1 level of theory, while the effects from the surrounding solvent environment were included using the fragment-based PE model. Additionally, for formaldehyde in water, we also considered a system where the two water molecules hydrogen-bonded to the formaldehyde are included in the QM subsystem and thus also treated at HF/pcseg-1 level of theory. The embedding potentials representing the solvents were obtained using the PyFraME Python package.^40^ Each solvent molecule is described by atom-centered multipoles, up to and including quadrupoles, atom-centered dipole–dipole polarizabilities, and a Lennard-Jones 6–12 potential. The multipoles and polarizabilities were derived using the LoProp scheme^41,42^ based on PBE0/6-31+G*^43−45^ calculations performed with the Dalton program package.^46,47^ The Lennard-Jones parameters were sourced from the GAFF force field,^48^ as provided by Van der Spoel et al.^49^ on https://virtualchemistry.org/, with the exception of water in the formaldehyde in water system, for which the CHARMM TIP3P Lennard-Jones parameters were applied.^50^

The geomeTRIC package^51^ was used together with the LSDalton program^46,52^ for the geometry optimizations. The environmental contributions for the QM/MM geometry optimizations were included using the FraME library.^53^ The small model systems were geometry optimized using full QM and QM/MM. Note that the solvent was frozen in all QM/MM optimizations.

The molecular properties of interest are the molecular Hessian, dipole gradients, and polarizability gradients (with incident light of 514.5 nm). These properties were calculated using LSDalton with the OpenRSP library,^54−56^ and using FraME to calculate environmental contributions. The IR and Raman intensities were derived from the dipole gradients and the polarizability gradients, respectively, as detailed by Dundas et al.^28^ Frequencies and normal modes were obtained from eigenanalysis of the Hessians.

### 2.2 Quantification of Pseudotranslation and Pseudorotation

In the following, we introduce a scheme to quantify pseudotranslational and pseudorotational contributions in normal modes in the QM/MM-PHVA approach. Like in standard vibrational analysis, the partial Hessian is mass-weighted and diagonalized, yielding 3*N* eigenvectors and eigenvalues. Then, the solute is centered by translating its center of mass to the origin of the coordinate system, and the three (or two if the molecule is linear) principal axes of inertia **I**_1_, **I**_2_, and **I**_3_ are determined. The principal axes of inertia are used to build vectors that describe rotation. The vectors corresponding to translations **D**_1_, **D**_2_, and **D**_3_ of the αth atom are defined as
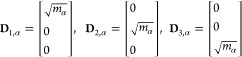
1where *m*_α_ is the atomic mass. The vectors corresponding to rotations **D**_4_, **D**_5_, and **D**_6_ are defined as
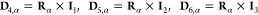
2where **R**_α_ is the position vector of the αth atom relative to the center of mass of all atoms. The normalized total vector **D**_*j*_ corresponding to a rotation or translation of the entire molecule consisting of *N* atoms can be written as
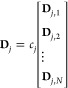
3where *c*_*j*_ is a normalization factor. In the case of linear molecules, **D**_6_ will not be generated. The pseudotranslational contributions in the *i*th normal mode are defined as
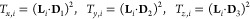
4where **L**_*i*_ is the *i*th normalized eigenvector of the mass-weighted Hessian and **D**_1_, **D**_2_, and **D**_3_ are the normalized vectors describing translation in *x*, *y*, and *z* directions, respectively. Similarly, the pseudorotational contributions in the *i*th normal mode are defined as
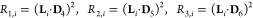
5where **D**_4_, **D**_5_, and **D**_6_ are the normalized vectors describing rotation around the principal axes of inertia. Since the vectors **D**_*j*_ are normalized and the eigenvectors **L**_*i*_ form an orthonormal basis, the following applies:
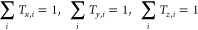
6
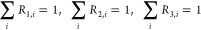
7

### 2.3 Hessian Comparison

We investigate the errors of the QM/MM-PHVA approach by comparing it to full QM calculations on the small model systems. For these systems, we calculated full QM Hessians, dipole gradients, and polarizability gradients, as well as partial QM/MM Hessians, dipole gradients, and polarizability gradients. Partial QM Hessians, dipole gradients, and polarizability gradients were extracted from the full QM quantities.

We used two schemes to compare the partial and full QM Hessians. The first scheme gives the contribution of environment molecules to the normal-mode eigenvectors of the full QM Hessian eigenvectors, which will not be present in the partial QM or QM/MM Hessians. To quantify this contribution, we divide each normalized eigenvector into two parts
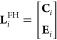
8where **C**_*i*_ corresponds to the core QM part (i.e., the solute) and **E**_*i*_ corresponds to the MM environment (i.e., the solvent). Then, we define the environmental contributions EC_*i*_ to the *i*th normal mode **L**_*i*_^FH^ as
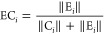
9with
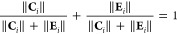
10

To identify which normal modes to compare from the full and partial QM Hessians, we calculated the overlap, i.e., the dot product, between the **C**_*i*_ vectors and the **L**_*i*_^PH^ eigenvectors of the partial QM Hessian. This shows the similarity between normal modes from the full and partial QM Hessians. The most similar normal modes were then used for the second scheme, where the directional deviation between the eigenvectors of the full and partial QM Hessians was calculated. For this, we define the angular deviation AD_*i*_ of the *i*th normal mode as
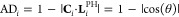
11Here θ is the angle between the normalized **C**_*i*_ and **L**_*i*_^PH^ vectors. The angular deviation was also used to compare the partial QM and QM/MM Hessians.

## 3 Results and Discussion

In the first part of this section, we investigate the pseudotranslational and pseudorotational contributions in the normal modes calculated using the QM/MM approach for the seven solute–solvent systems: furan in benzene, naphthalene in acetonitrile, pyridine in methanol, 1,3-butadiene in water, uracil in methanol, propanamide in water, and formaldehyde in water, including also the expanded QM subsystem for formaldehyde in water. We also investigate how the removal of the pseudotranslation and pseudorotation through projection, as described by Wilson et al.,^11^ affects frequencies, IR intensities, and Raman intensities of the remaining normal modes. Then, we turn toward a more detailed assessment of errors introduced, first, by the partial Hessian approximation and, second, by the use of QM/MM. To this end, we compare normal modes, frequencies, IR intensities, and Raman intensities obtained using the full and partial QM Hessians and the partial QM and QM/MM Hessians, respectively, of the five small model systems: pyridine in methanol, 1,3-butadiene in water, uracil in methanol, propanamide in water, and formaldehyde in water, including again the expanded QM subsystem for formaldehyde in water. In this context, we note that the eigenvectors and eigenvalues obtained from the PHVA are directly affected by the reduction of the full Hessian, whereas the dipole and polarizability gradients, and thus the IR and Raman intensities, are indirectly affected through the transformation from Cartesian to mass-weighted internal coordinates. The systems are summarized in Table 1. The seven large systems are used in the first part (Section 3.1), and the five small model systems are used in the second part (Sections 3.2 and 3.3).

### 3.1 Pseudotranslation and Pseudorotation

From a theoretical point of view, translation and rotation should not be projected out of the partial Hessian because, while some normal modes resemble translation and rotation, they are, in fact, collective vibrations between the QM and MM subsystems. We refer to those modes as pseudotranslation and pseudorotation. Instead of projecting out translation and rotation from the partial Hessian, a more effective approach is to identify modes with pseudotranslational and pseudorotational contributions that exceed a threshold and remove them since they are not well described.

In this study, we have found that the lowest-frequency modes of a partial Hessian will often be mixtures of internal motions of the QM subsystem with pseudotranslational and pseudorotational contributions. The projection results in a redistribution of the internal motion of the mixed modes to the other modes. This can alter the character of the affected modes.

**Figure 1:**
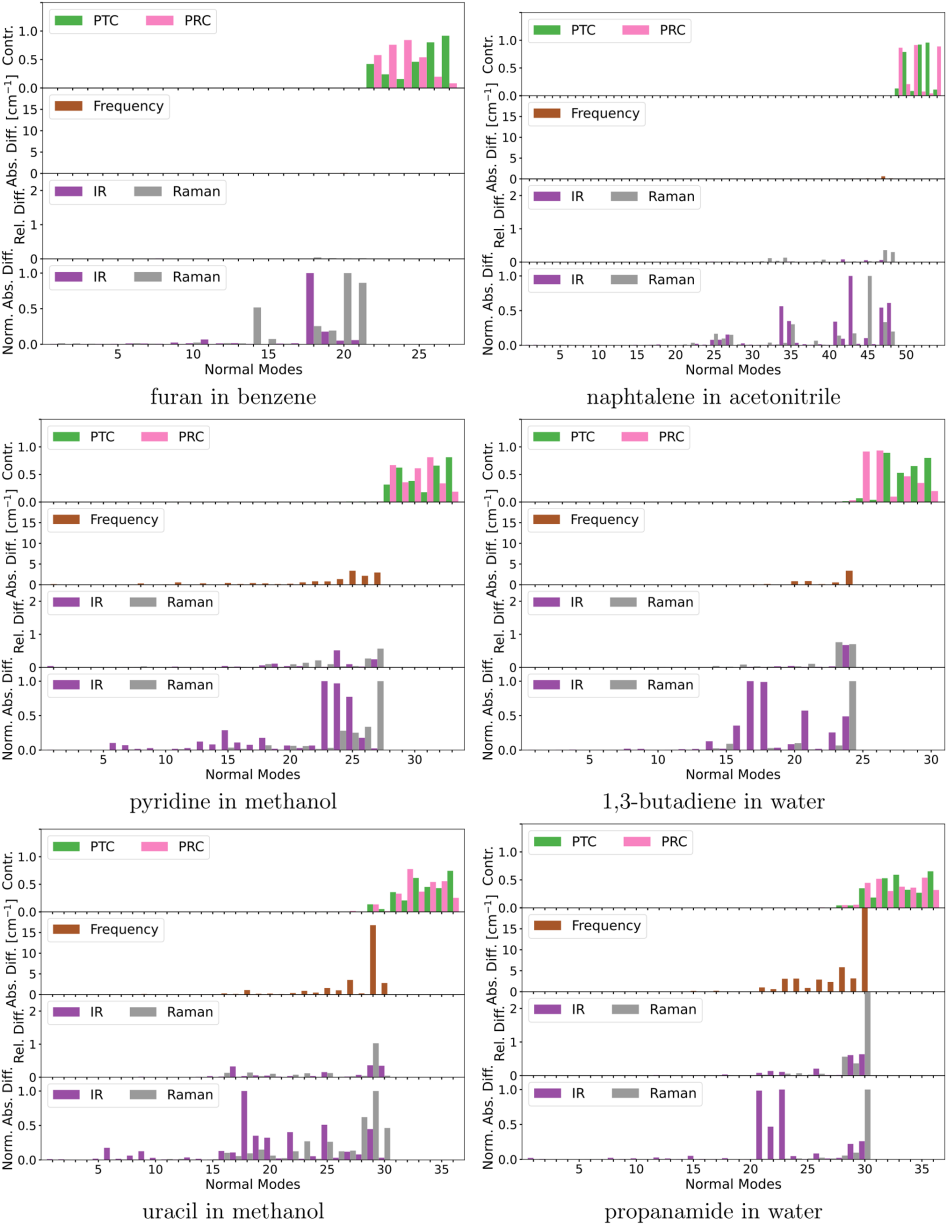
Identification and quantification of pseudotranslational contributions (PTC) and pseudorotational contributions (PRC) in normal modes of a series of solute–solvent systems and a comparison of frequencies, IR intensities, and Raman intensities before and after projecting out translation and rotation. Note that some bars are truncated due to scale limits.

**Figure 2:**
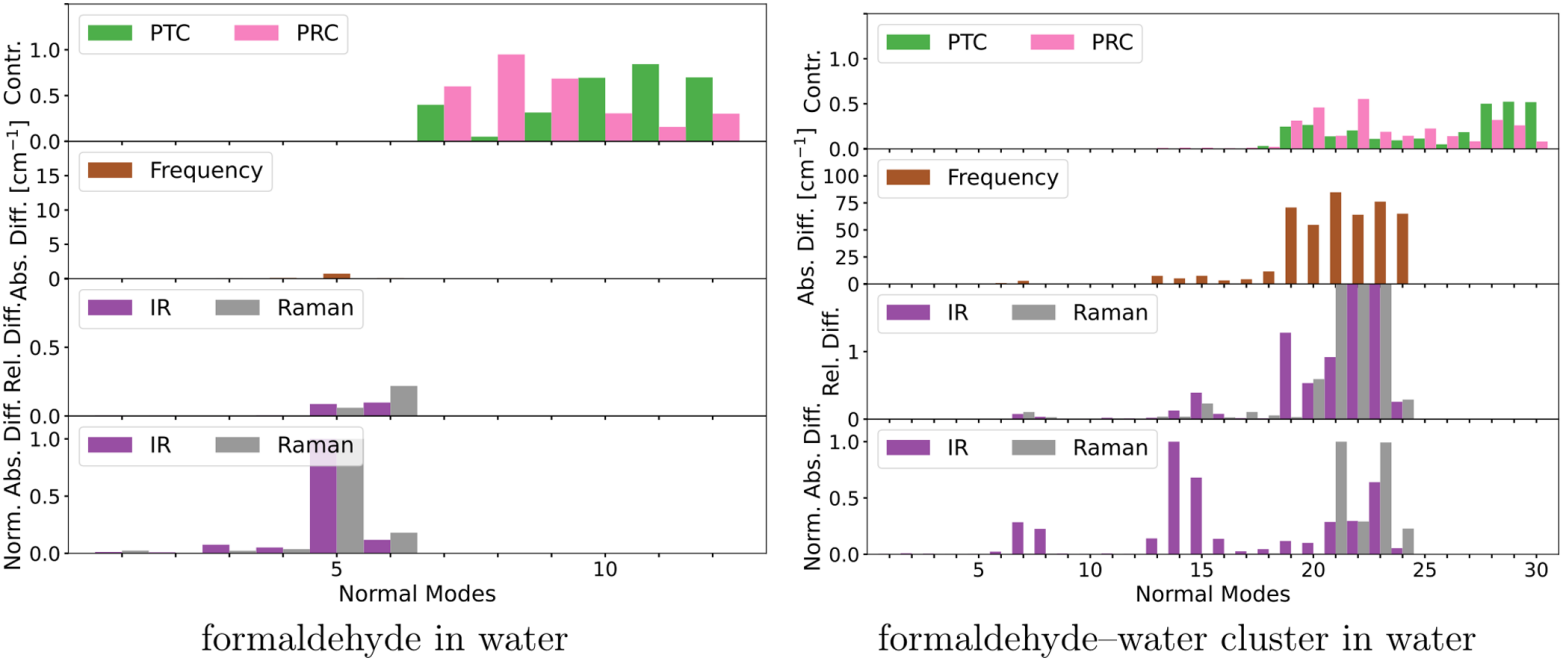
Identification and quantification of pseudotranslational contributions (PTC) and pseudorotational contributions (PRC) in normal modes of formaldehyde in water (left) without and (right) with the inclusion of two water molecules in the QM subsystem and thus in the partial QM/MM Hessian, as well as a comparison of frequencies, IR intensities, and Raman intensities before and after projecting out translation and rotation. Note that some bars are truncated due to scale limits.

Practically, the projection can be performed, and here we examine the effect on frequencies, IR intensities, and Raman intensities for the seven solute–solvent systems. The results for the six solute–solvent systems where only the solute molecule is in the QM subsystem are presented in Figure 1, while the results for formaldehyde in water with and without two solvent molecules in the QM subsystem are presented in Figure 2. Here, we investigate the absolute differences in frequencies, as well as the relative and normalized absolute differences in IR and Raman intensities. The normalized absolute differences refer to normalizing the absolute differences by the maximum absolute difference across all modes except the six lowest ones. Figures 1 and 2 also include the pseudotranslational and pseudorotational contributions to all normal modes calculated using eqs 4 and 5, respectively.

Of the six systems presented in Figure 1, the ones that are the least affected by the projection are furan in benzene and naphthalene in acetonitrile. In both cases, most of the pseudotranslational and pseudorotational contributions are in the six lowest-frequency modes. For furan in benzene, none of the other modes exceed pseudotranslational and -rotational contributions by more than 0.002%. A few modes in naphthalene in acetonitrile have pseudotranslational and pseudorotational contributions of around 0.3%. Although still small, the impact on the Raman intensities of those modes is substantial, about 30 to 35% relative difference while also having a considerable absolute difference. In both systems, the frequency is not altered appreciably by the projection.

In the cases of pyridine in methanol and 1,3-butadiene in water, the pseudotranslational and pseudorotational contributions for all modes except the six lowest-frequency modes go up to around 3%. The projection affects frequencies, IR intensities, and Raman intensities for both systems. The maximum frequency difference is around 3 cm^–1^, while the relative intensity differences are higher for pyridine in methanol (up to 76% for Raman and 68% for IR) than for 1,3-butadiene in water (up to 57% for Raman and 52% for IR). The absolute differences are also substantial for the aforementioned modes with high relative differences, so we expect to see a noticeable difference in the spectra for these systems.

Uracil in methanol and propanamide in water were affected the most by the projection. In both cases, there is at least one normal mode outside the six lowest-frequency modes with pseudotranslational and pseudorotational contributions exceeding 10%. For these normal modes, the projection substantially impacts the frequencies and intensities. Specifically for uracil in methanol, normal mode 29 has 14% each pseudotranslational and pseudorotational contributions, where the frequency changes by about 17 cm^–1^, the relative Raman intensity by about 103%, and the relative IR intensity by about 36%. For propanamide in water, normal mode 30 has 45% pseudorotational and 35% pseudotranslational contributions, where the change in frequency is about 129 cm^–1^, about 383% for the Raman intensity, and about 64% for the IR intensity. For the aforementioned normal modes with high relative differences, the absolute differences are also substantial and will thus lead to major differences in the spectra.

In Figure 2, we examine the effects of adding solvent molecules to the QM subsystem by comparing calculations on formaldehyde in water with and without two water molecules in the QM subsystem. In the following and in Figure 2, the latter is referred to as the formaldehyde–water cluster in water. The results for formaldehyde in water are consistent with the other solute–solvent systems presented in Figure 1. Specifically, pseudotranslational and pseudorotational contributions are observed only in the six lowest-frequency modes. For all other normal modes, the frequency errors are minimal, with the highest error being in normal mode 5, which exhibits a frequency error of around 1 cm^–1^. Notably, normal mode 5 also shows the greatest absolute difference in both IR and Raman intensities, with a relative difference of 9% for IR intensity and 6% for Raman intensity. For the formaldehyde–water cluster in water, the projection affects a significantly larger number of normal modes beyond the six lowest-frequency modes. The majority of pseudotranslational and pseudorotational contributions are concentrated in the 13 lowest-frequency modes, each of which shows contributions of at least 10%. However, up to the 18 lowest-frequency modes exhibit at least 1% pseudotranslational and pseudorotational contributions. On most of the 18 lowest-frequency modes, the projection has a considerable impact on frequencies and intensities. Specifically, modes 19–24 exhibit substantial frequency errors ranging from 55 to 85 cm^–1^, all corresponding to intermolecular stretching or bending modes in the formaldehyde–water cluster. Interestingly, the normal modes in the formaldehyde–water cluster that have the greatest overlap with the normal modes in formaldehyde in water are among the 13 highest-frequency modes, which exhibit similarly low errors as those in formaldehyde in water. Therefore, the increase in pseudotranslational and pseudorotational contributions and the associated errors seen in the formaldehyde–water cluster likely stem from the inclusion of the two solvent water molecules to the QM subsystem.

This analysis shows that projecting out translation and rotation can substantially affect frequencies, IR intensities, and Raman intensities. Generally, the normal modes most affected are the low-frequency ones, while the high-frequency modes are unaffected. It is also noteworthy that the IR and Raman intensities are more sensitive than the frequencies. For example, for a normal mode in 1,3-butadiene in water, the difference was more than 50% while the frequency only changed by about 3 cm^–1^. Moreover, by including solvent molecules in the QM subsystem, the pseudotranslational and pseudorotational contributions are affecting more normal modes compared to systems without solvent molecules. In the systems where only the solute is in the QM subsystem, these contributions are visible in, at most, nine of the lowest-frequency modes. Clearly, the impact of projection can be substantial, and since it is also unfounded, a better approach to eliminating the problematic modes is to quantify the pseudotranslational and pseudorotational contributions and remove the modes with contributions higher than a threshold.

### 3.2 Comparison between Full and Partial QM Hessians

**Figure 3:**
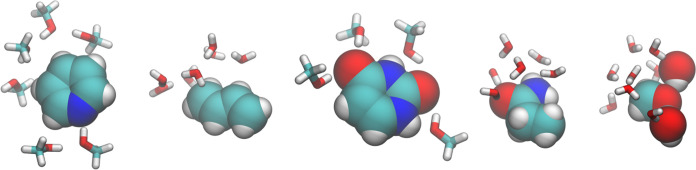
Geometries of the five small model systems: pyridine in methanol, 1,3-butadiene in water, uracil in methanol, propanamide in water, and formaldehyde in water. QM molecules are depicted as van der Waals spheres and MM molecules as sticks. For formaldehyde in water, we only depict the formaldehyde–water cluster in water.

**Figure 4:**
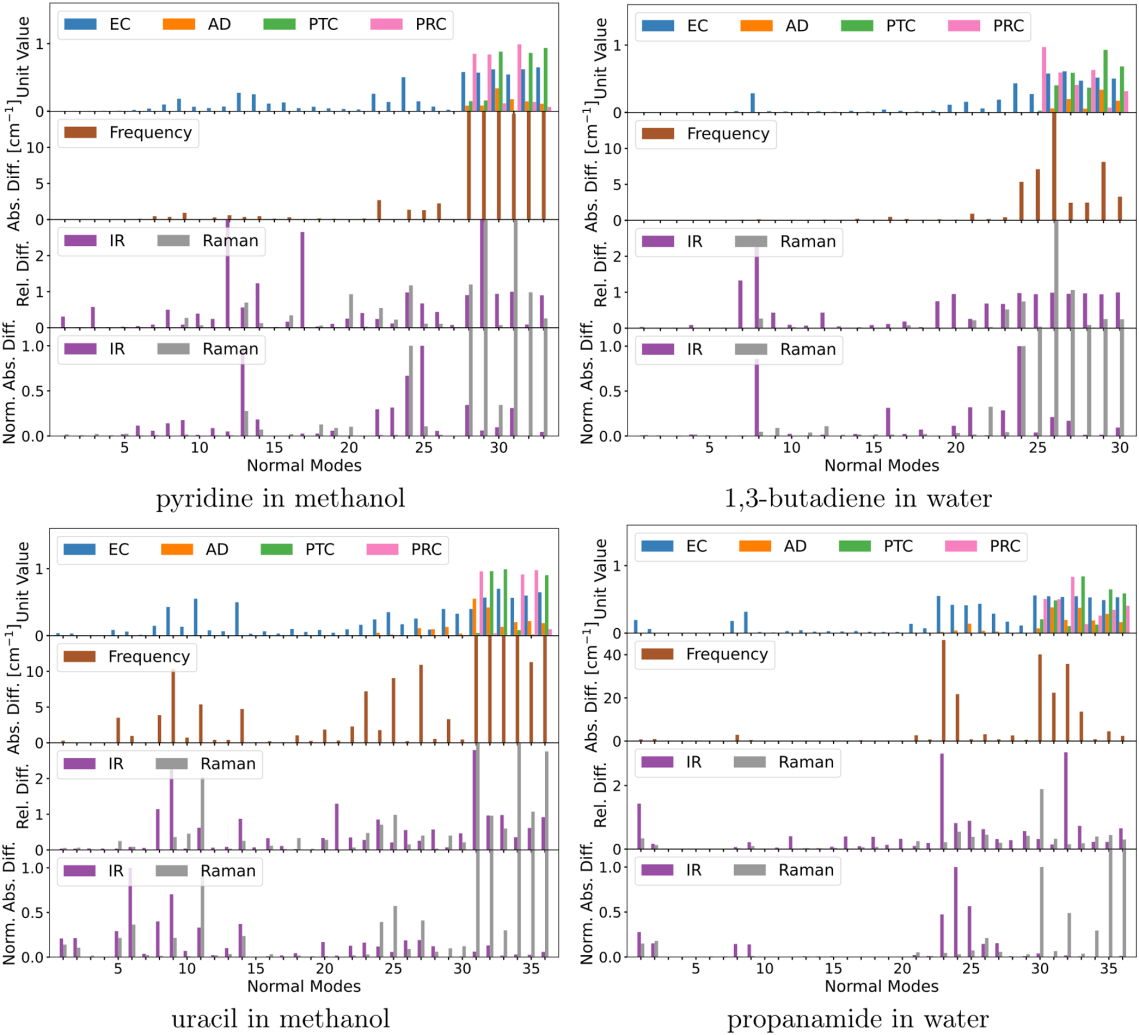
Comparison of normal modes obtained from full and partial QM Hessians of small model systems. Depicted are the environmental contributions (EC), the angular deviations (AD), the pseudotranslational contributions (PTC), the pseudorotational contributions (PRC), the frequency change, and the IR and Raman intensity changes. Note that some bars are truncated due to scale limits.

**Figure 5:**
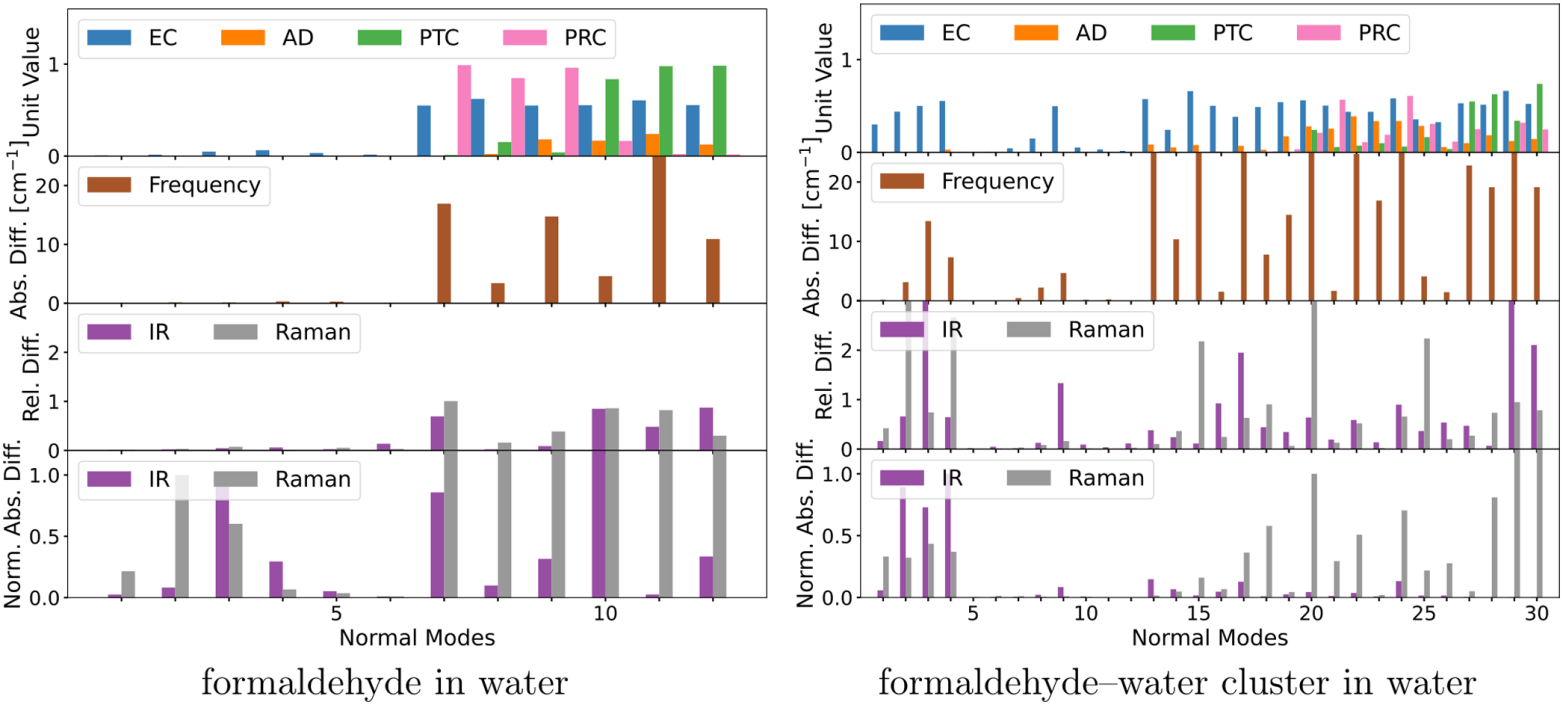
Comparison of normal modes obtained from full and partial QM Hessians of formaldehyde in water (left) without and (right) with the inclusion of two water molecules in the partial QM Hessian. Depicted are the environmental contributions (EC), the angular deviations (AD), the pseudotranslational contributions (PTC), the pseudorotational contributions (PRC), the frequency change, and the IR and Raman intensity changes. Note that some bars are truncated due to scale limits.

To investigate the errors introduced by the PHVA approximation itself, i.e., not including errors from QM/MM, we compare the full and partial QM Hessians as well as the frequencies, IR intensities, and Raman intensities derived using these Hessians. To keep the computational cost low, we used the small model systems pyridine in methanol, 1,3-butadiene in water, uracil in methanol, propanamide in water, and formaldehyde in water (see Figure 3). For the latter, we also include the formaldehyde–water cluster in water, i.e., where the two water molecules hydrogen-bonded to formaldehyde are included in the partial QM Hessian. We compared the absolute errors in frequencies and relative and normalized absolute errors in the IR and Raman intensities of the normal modes with a high similarity according to the method described in Section 2.3. The normalized absolute error is calculated as the absolute difference divided by the maximum absolute difference of all normal modes except the six lowest-frequency modes. In addition to the pseudotranslational and pseudorotational contributions, we also calculated the environmental contribution (eq 9) and angular deviation (eq 11). The environmental contribution is a measurement of the degree to which molecules in the solvent environment are involved in a given normal mode, while the angular deviations are a measure of the directional similarity between the vibrations in the solute before and after applying the PHVA approximation. The results are presented in Figures 4 and 5.

As expected, normal modes with substantial pseudotranslational and pseudorotational contributions also include a high environmental contribution and angular deviation. For the systems without solvent molecules in the partial Hessian, the pseudotranslational and pseudorotational contributions appear in the six lowest-frequency modes, except in the case of propanamide in water, where there are also contributions in the seventh-lowest frequency mode. For the formaldehyde–water cluster in water, the 12 lowest-frequency modes have non-negligible pseudotranslational and pseudorotational contributions. These normal modes are not well described within the PHVA approximation, which is also reflected in the high errors in frequencies and intensities. The following discussion does not consider the normal modes with high pseudotranslational and pseudorotational contributions.

To better understand the errors in the intensities due to the PHVA approximation, we need to consider the truncation of the dipole and polarizability gradients, i.e., the removal of the derivatives with respect to the positions of nuclei in the solvent environment, as well as the fact that the gradients are transformed using the transformation matrix obtained from the partial Hessian. Hence, the origin of the intensity errors can come from the truncation of the Hessian and the truncation of the gradients. The total impact is reflected by the relative intensity differences. Since the environmental contributions are correlated to the expected changes in the transformation matrix, it is possible to identify for some modes if the high relative intensity difference is dominated by the truncation of the Hessian or by the truncation of the gradients. It is important to emphasize that we also consider the normalized absolute intensity difference, which is highest for the largest peak in the spectrum and expected to be considerably large for other intense peaks in the spectrum. Our focus lies on considering cases where both the normalized absolute intensity differences and the relative intensity differences are substantial, as this indicates a noticeable difference in the final spectrum.

For pyridine in methanol, the frequencies are largely unaffected in the high-frequency part of the spectrum and with minor errors in the low-frequency part. The maximum error is around 3 cm^–1^. The impact on IR and Raman intensities, on the other hand, is substantial. In particular, the IR intensities of normal modes 13, 14, 24, and 25 and the Raman intensity of normal mode 24 have considerable relative and absolute errors. These four normal modes represent in-plane bending motions of the pyridine molecule, which couple to the C–O stretching mode of methanol in the case of modes 13 and 14 and to the torsion mode of methanol in the case of modes 24 and 25. These modes have environmental contributions that are removed through the PHVA approximation, which explains the resulting frequency errors and, to some degree, the intensity errors. Similar to the normal modes with high errors, mixing between solute and solvent vibrations is also observed in other modes, yet the associated frequencies and intensities have low errors. For example, in normal mode 9, a mixing between an in-plane bending mode in pyridine and the O–H rocking mode in methanol can be observed. However, since normal mode 9 has a low intensity in both the partial and full treatment, the absolute error introduced to the IR intensities is also low. The degree of environmental contribution is similar to the other aforementioned modes with higher errors, but the relative intensity errors are small. Therefore, for this particular mode, the errors must be dominated by the truncation of the dipole and polarizability gradients, which, however, does not have a strong impact on the final intensities.

For 1,3-butadiene in water, the errors in the frequencies are also minor, except for mode 24, where the error is about 5 cm^–1^. The errors in IR and Raman intensities are primarily in the low-frequency end of the spectrum, where especially normal mode 24 stands out with large relative and absolute errors for both IR and Raman intensities. In the high-frequency part of the spectrum, only the IR intensity of normal mode 8 has substantial relative and absolute errors. Both of these modes have high environmental contributions. Normal mode 8 includes C=C stretching in 1,3-butadiene that couples to O–H bending in some water molecules, while normal mode 24 includes rotation around the central C–C bond in 1,3-butadiene and rotation of some water molecules. Notably, both IR and Raman intensities for normal mode 24 become much smaller in the PHVA. This implies that the water rotation contributes much stronger to the IR and Raman intensities than the 1,3-butadiene deformation vibration around the central C–C bond.

In contrast to the other solute–solvent systems, the normal modes of uracil in methanol have much higher frequency errors across the entire spectrum. The frequencies of normal modes 9, 23, 25, and 27 are close to 10 cm^–1^, while the frequencies of modes 5, 8, 11, 14, and 29 are around 5 cm^–1^. All of these normal modes have clear environmental contributions, and some low-frequency modes also have angular deviations. However, it is worth noting that the size of the environmental contributions does not necessarily correlate with the size of the errors. For example, the environmental contributions are rather small for normal mode 5, yet the frequency error is substantial. The IR intensity errors are most pronounced in normal modes 8, 9, 11, and 14, where the large environmental contributions come from O–H rocking motions of methanol that are mixed with in-plane bending modes of uracil. The Raman intensity errors are highest in normal modes 11, 24, and 25, where the two latter modes correspond to in-plane bending motions of uracil mixed with torsion motions of methanol. An explanation for the frequency errors that appear across the entire frequency range is that uracil, with its two carbonyl and two amide groups, provides two hydrogen-bond acceptors and two hydrogen-bond donors. The PHVA approximation substantially affects the description of the coupling between the atoms involved in the hydrogen bonds, which are involved in several normal modes across the entire frequency range.

The frequency errors for propanamide in water are generally small, but two modes stand out with high errors, namely modes 23 and 24, with errors of about 45 and 20 cm^–1^, respectively. The intensity errors are mostly prominent in the lower-frequency normal modes, except for normal mode 1. The largest IR intensity errors are observed for normal mode 1, 23, 24, and 25. The Raman errors are low overall. A closer look at normal modes 23, 24, and 25 reveals that all three modes consist of NH_2_ wagging motions mixed with rotations of some water molecules. Normal mode 1, on the other hand, is a mixture of the NH_2_ asymmetric stretching mode in propanamide and the asymmetric stretching mode of some water molecules. All of these modes have substantial environmental contributions, and the low-frequency modes also have angular deviations. There are also normal modes with clear environmental contributions but small errors overall. This applies to normal modes 8 and 9, which correspond to NH_2_ bending and C=O stretching motions of propanamide mixed with water bending motions. Both modes result in a high IR intensity in both the full and partial counterparts, which indicates that the water bending contributes little to the IR intensity of these modes.

For formaldehyde in water (results presented in Figure 5), the frequency errors are generally small, approximately 1 cm^–1^. Similarly, the errors are also small for the normal modes local to the formaldehyde of the formaldehyde–water cluster, i.e., normal modes that have substantial overlap with the normal modes of formaldehyde in water, which is normal modes 5, 6, 7, 10, 11, and 12 in the formaldehyde–water cluster. These modes have small to no environmental contributions. Moreover, they do not exhibit substantial errors in absolute or relative IR and Raman intensities. Conversely, normal modes 1, 2, 3, 4, 8, and 9 in the formaldehyde–water cluster involve the stretching and bending of the two water molecules. Modes 13–19 correspond to intermolecular vibrations in the QM subsystem, with modes 13–18 corresponding to intermolecular bending modes and mode 19 representing an intermolecular stretching mode. Normal modes involving the two water molecules in the formaldehyde–water cluster show noticeable environmental contributions. The largest frequency errors (40–50 cm^–1^) are observed in the intermolecular bending modes 13, 15, and 17, while the most considerable errors in IR and Raman intensities are found in the water stretching modes 2, 3, and 4. Notably, modes 17 and 18 also exhibit substantial relative and absolute Raman intensity errors. It is expected that molecular vibrations of the two water molecules are strongly coupled to the motions of other water molecules due to hydrogen bonding, thus potentially having a substantial effect on all intramolecular and intermolecular modes involving water in the partial Hessian. Interestingly, the carbonyl stretching mode of formaldehyde remains well-described both with and without including water molecules in the partial Hessian.

Generally, for normal modes consisting of local vibrational motions in the solute and solvent separately, using the PHVA approximation does not have a substantial effect. Conversely, if a normal mode involves collective motions of atoms in the solute and solvent, the PHVA approximation may introduce large errors. This becomes especially apparent in the formaldehyde–water cluster, where the molecular vibrations of the two water molecules in the cluster are strongly coupled to the water molecules in the solvent. Although the environmental contribution does not distinguish between the two cases, large environmental contributions typically indicate large frequency errors due to the lack of coupling between solute and solvent atoms in the partial Hessian. However, as seen in mode 5 for uracil in methanol, the environmental contribution can be small while large errors still occur. Conversely, there are also instances, e.g., normal modes 8 and 9 of propanamide in water, with clear environmental contributions, yet the errors are relatively small. The intensity errors, on the other hand, are typically dominated by the truncation of the dipole and polarizability gradients. The impact of the truncated properties is shown by the relative intensity differences. Together with the environmental contributions, which indicate the errors of the transformation matrix, it becomes apparent for some normal modes if it is the truncation of the Hessian that dominates or the truncation of the dipole and polarizability gradients. In the cases investigated here, there are many normal modes with a high relative intensity error and low environmental contributions. Thus, the intensity errors are mostly dominated by the truncation of the dipole and polarizability gradients. The angular deviation is only visible in the low-frequency part of the spectrum and is generally negligible in the rest of the spectrum, indicating that the molecular vibrations in the solute do not change substantially. Although it cannot be generalized without further investigation, for our test cases, the IR intensities of the high-frequency modes were more affected than the Raman intensities. Simultaneous large relative and absolute errors for the Raman intensities are only observed in a few cases. For our systems, it is to some degree due to the higher number of high-intensity normal modes in IR compared to Raman. Also, it is worth noting that the solvent environment contributes to some high-frequency modes that are usually considered to be very local, e.g., normal mode 1 of propanamide in water. Hence, one cannot assume that all high-frequency modes will not be affected by the PHVA approximation. The inclusion of water molecules in the partial Hessian of the formaldehyde in water system did not impact the local modes of the formaldehyde but resulted in high errors in the delocalized normal modes corresponding to intermolecular modes within the formaldehyde–water cluster and in the normal modes of the two included water molecules.

### 3.3 Comparison between Partial QM and QM/MM Hessians

**Figure 6:**
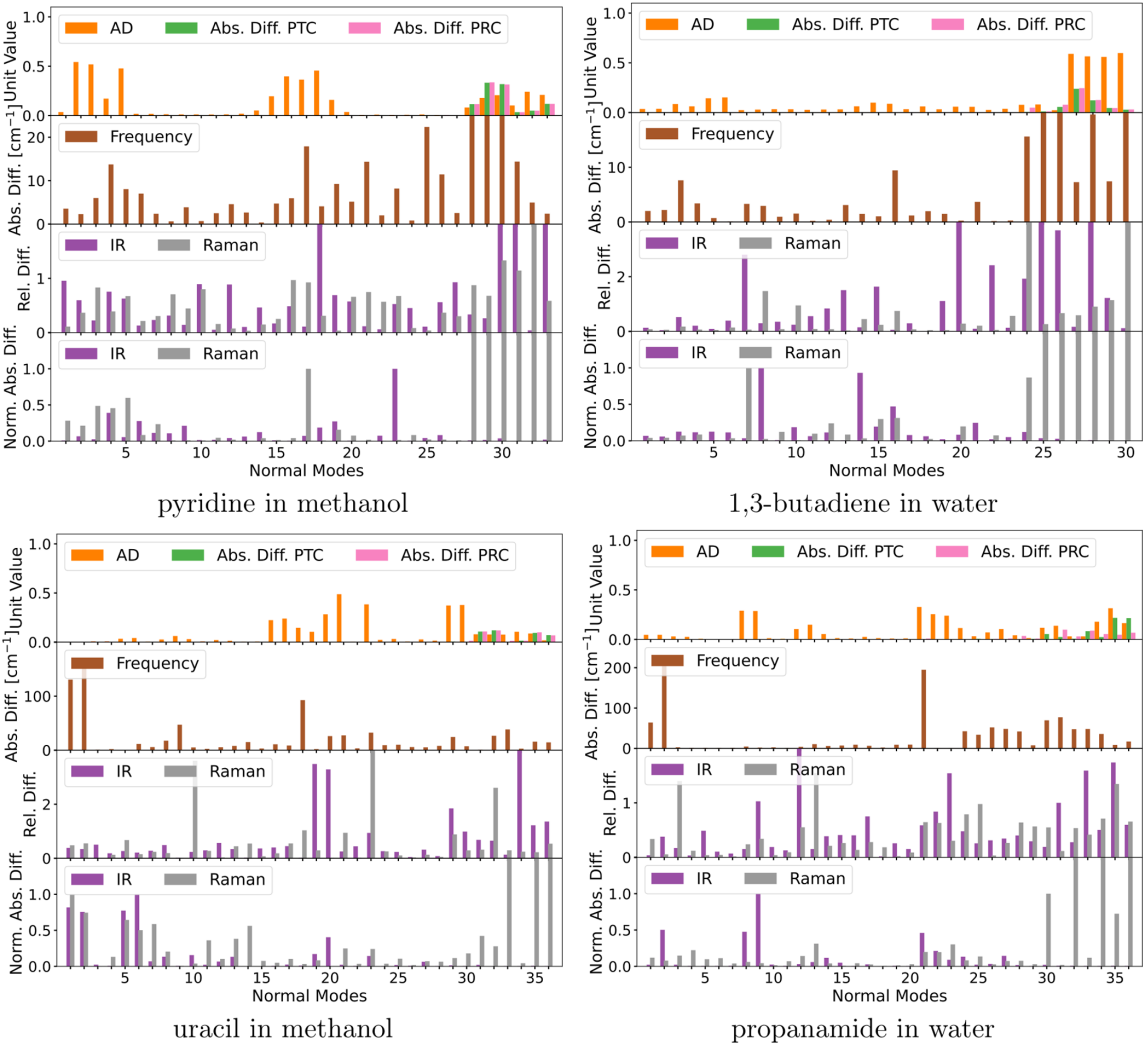
Comparison of normal modes obtained from the partial QM and QM/MM Hessians of small models systems. Depicted are the angular deviations (AD), the absolute difference in pseudotranslational contributions (PTC), the absolute difference in pseudorotational contributions (PRC), the frequency change, and the IR and Raman intensity changes. Note that some bars are truncated due to scale limits.

**Figure 7:**
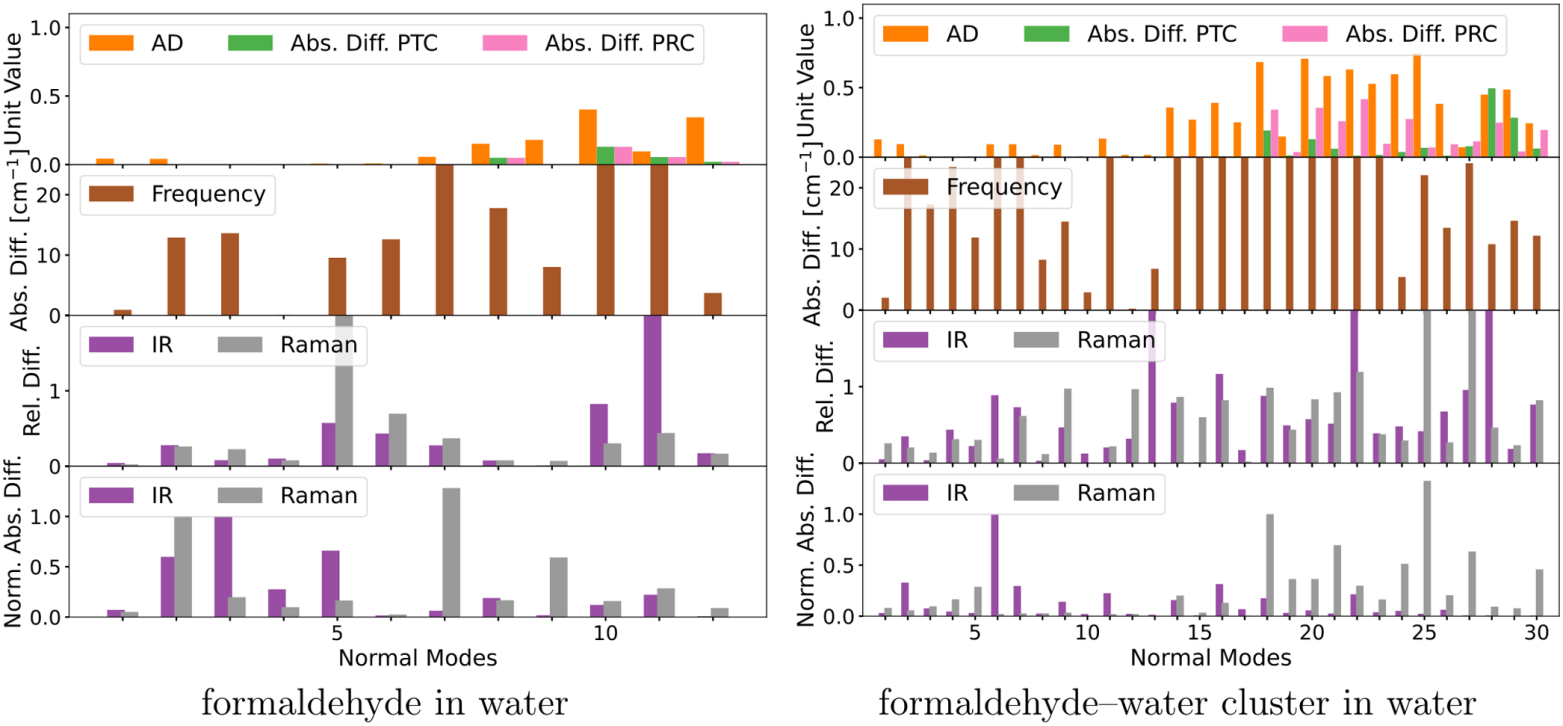
Comparison of normal modes obtained from the partial QM and QM/MM Hessians of formaldehyde in water (left) without and (right) with the inclusion of two water molecules. Depicted are the angular deviations (AD), the absolute difference in pseudotranslational contributions (PTC), the absolute difference in pseudorotational contributions (PRC), the frequency change, and the IR and Raman intensity changes. Note that some bars are truncated due to scale limits.

Here, we investigate the differences between the normal modes obtained from the partial QM and QM/MM Hessians. The partial QM Hessian is considered the reference for the partial QM/MM Hessian. The errors observed here are thus in addition to those found in the previous section. However, it is possible that there is a cancellation of errors. We look at the angular deviation between the normal modes, the absolute difference in pseudotranslational and pseudorotational contributions, the absolute difference in frequencies, and the relative and normalized absolute difference in IR and Raman intensities. The results of this investigation are shown in Figures 6 and 7. The QM/MM geometries were obtained from a constrained QM/MM geometry optimization of the fully optimized structures in Figure 3. To check the similarity between the QM- and QM/MM-optimized structures, we calculated the root-mean-square deviations (RMSDs) of the nuclear positions. The RMSDs are 0.20 Å for pyridine in methanol, 0.31 Å for 1,3-butadiene in water, 0.02 Å for uracil in methanol, and 0.06 Å for propanamide in water. The structural differences are thus larger for pyridine in methanol and 1,3-butadiene in water than for propanamide in water and uracil in methanol. Since the solvent environments are frozen, the RMSDs are from the solute only. The change in geometry can be internally in the solute and displacements of the solute as a whole within the solvent cage. For formaldehyde in water, the RMSD is 0.05 Å. In the formaldehyde–water cluster in water, the RMSD increases to 0.21 Å, which includes the contribution from changes in the geometry of the two water molecules. When the water molecules in the formaldehyde–water cluster are not considered, the RMSD for formaldehyde alone is 0.05 Å. Additionally, the RMSD between the formaldehyde molecules in the two QM/MM systems is 0.04 Å.

For pyridine in methanol, the errors in the frequencies are mostly below 10 cm^–1^, apart from those of normal modes 4, 17, 21, 25, and 26 that go above. The IR intensities associated with normal modes 4 and 23 have high relative and absolute errors, and the same applies to the Raman intensities of normal modes 3, 4, 5, and 17. The change in pseudotranslational and pseudorotational contributions occurs only in the six lowest frequency modes. The angular deviations are relatively high for the high-frequency modes 2, 3, 4, and 5, as well as for the lower-frequency modes 15, 16, 17, 18, and 19. This could explain the errors observed for those modes. Based on our results, we are not able to quantify to which degree the angular deviations stem from structural or property differences.

Similarly, for butadiene in water there are errors in the frequencies of most normal modes, although they are overall lower. For normal modes 3, 16, and 24, the frequency error is around 10 cm^–1^, while high relative and absolute errors are found for the IR intensity of normal mode 15 and the Raman intensities of normal modes 16 and 24. The partial QM/MM Hessian results in a small pseudorotational contribution in normal mode 24, i.e., the seventh-lowest frequency mode. This may explain the high frequency and Raman intensity errors associated with this mode. The angular deviations are relatively small for all normal modes except the four lowest frequency modes. This is surprising when considering that the RMSD between the QM and QM/MM geometries is larger than the one for pyridine in methanol, where the angular deviations were substantially larger.

The normal modes of both uracil in methanol and propanamide in water have large frequency errors. Especially modes 1, 2, and 18 in uracil in methanol and modes 1, 2, and 21 in propanamide in water have errors close to or above 100 cm^–1^. These large errors seem to be related to the fact that all of the modes include vibrations of an N–H moiety that is involved in hydrogen bonds to solvent molecules. Hydrogen bonds crossing the QM–MM boundary are only partially described within the QM/MM model. This is also reflected through the change in the N–H bond length that increases by 0.015 Å for uracil in methanol and 0.017 Å for propanamide in water when going from full QM to QM/MM, indicating too strong hydrogen bonds. Additionally, the frequency errors are likely made worse by basis-set superposition errors in the reference calculations. On the other hand, the normal modes that include C=O stretching, i.e., modes 5 and 6 in uracil in methanol and modes 8 and 9 in propanamide in water, which are also involved in hydrogen bonds, have relatively low errors. Unlike the N–H bond length, the C=O bond length does not change substantially in uracil in methanol. In propanamide in water, the C=O bond is elongated by 0.012 Å. This is in line with the difference in angular deviations of the modes. The lower errors of the C=O stretching modes compared to the N–H stretching suggest a higher sensitivity for hydrogen-bond donors than for hydrogen-bond acceptors. In regards to the pseudotranslational and pseudorotational contributions, although small, there are changes outside of the six lowest frequency modes. For example, for normal mode 30 for uracil in methanol and normal modes 27, 28, 29, and 30 for propanamide in water. Except for normal mode 30 for propanamide in water, all of these modes had no contributions in the partial QM Hessian and, therefore, arose solely from the partial QM/MM Hessian.

In the comparison of formaldehyde in water and the formaldehyde–water cluster in water, we focus on the local vibrational modes of formaldehyde as those are the ones of main interest. The normal modes of formaldehyde in water are asymmetric C–H_2_ stretching (mode 1), symmetric C–H_2_ stretching (mode 2), C=O stretching (mode 3), C–H_2_ scissoring (mode 4), C–H_2_ rocking (mode 5), and C–H_2_ wagging (mode 6) which in the formaldehyde–water cluster correspond to normal modes 4, 5, 8, 10, 12, and 13, respectively.

The asymmetric C–H_2_ stretching mode of formaldehyde in water is reproduced well by the QM/MM-PHVA with minor frequency and intensity errors. Increasing the size of the QM subsystem results in a larger frequency error of 23 cm^–1^ and also larger but minor relative and absolute Raman intensity errors. In the symmetric C–H_2_ stretching mode, the frequency error is slightly reduced from 13 to 12 cm^–1^. In contrast, the improvement of the C=O stretching mode is more substantial. Here the frequency error is lowered from 14 to 8 cm^–1^. The intensities are also substantially improved in terms of normalized absolute difference, but the relative errors are small in both systems, so it is not likely to be visible in a spectrum. The C–H_2_ scissoring mode is well described in both systems but slightly worse in the formaldehyde–water cluster, while the C–H_2_ rocking and wagging modes are reproduced better using the expanded QM subsystem. For the scissoring mode, the frequency error goes up from 0 to 3 cm^–1^, but intensities are slightly improved. In the rocking and wagging modes, the frequency errors are reduced from 10 to 0 cm^–1^ and 13 to 7 cm^–1^, respectively, and the intensities of the rocking mode are also improved.

Overall, the partial QM/MM PHVA approach increases the pseudotranslational and pseudorotational contributions to other low-frequency modes outside of the six lowest ones. Thus, it becomes more important to investigate the pseudotranslation and pseudorotational contributions. The angular deviations are considerable for all systems in different parts of the frequency spectrum. Overall, the intensities obtained from the QM PHVA are largely reproduced by the QM/MM PHVA. Substantial errors were only observed for a few modes. Concerning frequencies, the errors are mostly below 10 cm^–1^, but in some cases they are higher. Very high errors of 100 to 200 cm^–1^ are observed for normal modes involving hydrogen-bond donors, such as those in uracil in methanol and propanamide in water. For the formaldehyde in water system, expanding the QM subsystem to include the two hydrogen-bonded water molecules led to improvements in some vibrational modes but also had adverse effects on others. Notably, the C=O stretching mode was better reproduced, which is expected given the enhanced interaction with the solvent environment. The adverse effects could be due to an imbalanced description of the solvent environment. Thus, to gain an overall improved description, it would be preferable to enclose the solute with additional QM solvent molecules.

## 4 Conclusions

In this work, we analyzed the accuracy and applicability of the PHVA approximation when used with QM/MM for solute–solvent systems with small, rigid solutes. To assess the errors, we examined the normal modes of seven different systems and their associated frequencies, IR intensities, and Raman intensities. For one of the systems, we also investigated the effects of expanding the QM subsystem by including solvent molecules.

First, we addressed how to identify vibrational modes of the QM subsystem that include relative motions between the QM and MM subsystems and can be characterized as pseudotranslation and pseudorotation. This revealed that up to nine of the lowest-frequency modes were affected for the systems where the QM subsystem only included the solute. For the system with an expanded QM subsystem, 13 normal modes were affected, indicating that larger and more flexible QM subsystems are more prone to experiencing pseudotranslational and pseudorotational contributions in their normal modes. This suggests that as the QM subsystem is expanded to include more solvent molecules, the coupling between the QM and MM subsystems becomes more pronounced, leading to additional modes being influenced by these mixed motions. As a result, careful attention must be paid to properly identifying and removing these pseudotranslational and pseudorotational modes to avoid misinterpreting the vibrational spectrum. We also investigated the impact of removing the pseudotranslational and pseudorotational contributions by projecting out translation and rotation. This will remove the pseudotranslational and pseudorotational contributions, but since the modes are usually mixed modes, any internal motion of the affected modes is redistributed to other modes. Thus, some normal modes can be adversely affected by the projection. Instead of projecting out translation and rotation, the normal modes with substantial pseudotranslational and pseudorotational contributions should be removed.

In the next analysis, we investigated the errors that are introduced through the PHVA approximation by comparing full and partial QM Hessians. This comparison revealed that if the environment participates in a normal mode, the errors in frequencies due to the PHVA approximation generally increase. However, with respect to the errors in the intensities, the truncation of the dipole and polarizability gradients was mostly the dominating factor rather than the transformation to normal coordinates using an approximate transformation matrix. Thus, there were cases where the environment did not contribute particularly strongly to a normal mode, but still substantial changes in the intensity were observed. Regarding the angular deviations, they were mainly observed in the low-frequency part of the spectrum. Thus, the vibrational motions of the QM subsystem in the other parts of the spectrum were largely unaltered. Although the environmental contributions were also most prominent in the low-frequency part, there were, in some cases, also substantial contributions to the higher-frequency modes. The expanded QM subsystem did not have any negative effect on the local normal modes of the solute compared to the nonexpanded QM subsystem.

Finally, we investigated the errors that are introduced by the QM/MM PHVA compared to the QM PHVA. Here, we observed very large errors in the frequencies of normal modes involving hydrogen-bond donors that hydrogen bond with solvent molecules in the environment. The intensities were less affected overall in terms of having simultaneously large relative and absolute errors. Only a few normal modes had substantial intensity errors. An additional source of error introduced by the QM/MM PHVA is the structural differences, which we quantified by calculating the root-mean-square deviations (RMSD) of nuclear positions. Although larger RMSDs could be observed for two of the systems, which most likely contributed substantially to the overall errors of these systems, they were not necessarily the most critical factor since the systems with low RMSDs had higher errors than the ones with high RMSDs. The angular deviations revealed that many normal modes from the QM/MM Hessians were dissimilar in direction compared to the normal modes from the partial QM Hessians, potentially due to the aforementioned difference in geometries. Nonetheless, we were not able to find a clear correlation between the errors and the angular deviations. Our results based on calculations using an expanded QM subsystem show that while some vibrational modes improved, others were negatively affected, most likely due to an imbalanced description of the solvent environment. Including more QM solvent molecules may enhance overall accuracy.

In summary, our investigations give insights into the errors in the vibrational frequencies and associated IR and Raman intensities when using the PHVA approximation both for full QM and QM/MM approaches. Notably, the errors were more pronounced in the low-frequency part of the spectrum, but they can also be substantial in the higher-frequency parts. Additionally, we show how to identify normal modes with high pseudotranslational and pseudorotational contributions so that they can be removed since they are poorly described within the PHVA approximations. Lastly, with regard to the QM/MM scheme, our work shows that hydrogen-bonded solvent molecules should be included in the QM subsystem to avoid very large errors. In particular, when there are hydrogen-bond donors in the QM subsystem.

## Data Availability

The data presented in this paper is available at https://doi.org/10.5281/zenodo.12684650
